# Evaluating a rapid fit-testing method for N95 respirators in Chinese healthcare workers: a paired crossover simulation and diagnostic agreement study

**DOI:** 10.3389/fpubh.2026.1911331

**Published:** 2026-07-23

**Authors:** Guo Jing Han, Lina Li, Ru Fan, Wei Li, Zhao Jun Sheng, Wei Hua Zhang

**Affiliations:** 1 Department of Respiratory and Critical Care Medicine, the First Medical Centre of Chinese PLA General Hospital, Beijing, China; 2 Beijing SINOVO Times Technology Co. Ltd., Beijing, China; 3 Department of Respiratory and Critical Care Medicine, the Eighth Medical Center of Chinese PLA General Hospital, Beijing, China

**Keywords:** chest compressions, fit testing, healthcare workers, N95 respirators, rapid-test protocol

## Abstract

**Background:**

China lacks standardized rapid fit-testing protocols for N95 respirators. This exploratory study assessed the feasibility between the OSHA 2.5-min quantitative fit-testing protocol and the 6-min Chinese reference protocol among Chinese healthcare workers, also assessing mask seal degradation during chest compressions.

**Methods:**

A total of 54 nurses and physicians were recruited for this paired crossover simulation and diagnostic agreement study. Participants underwent quantitative fit testing using two N95 respirator models: D918 (a foldable respirator) and D920 (a cup-type respirator). Fit-test outcomes obtained using the 2.5-min protocol were compared with those obtained using the 6-min reference protocol. Paired pass results were analyzed using McNemar’s test, and agreement was assessed using Cohen’s kappa with 95% confidence intervals. Diagnostic indices, including sensitivity, specificity, positive predictive value, negative predictive value, and likelihood ratios, were calculated from raw 2 × 2 contingency tables. Seal integrity during simulated chest compressions was also evaluated.

**Results:**

Using the 6-min protocol as the reference, the D918 respirator showed pass rates of 75.93% [95%CI, 0.63 to 0.85] for the 2.5-min protocol and 81.48% [95%CI, 0.69 to 0.90] for the 6-min protocol. The corresponding sensitivity, specificity, PPV, PLR, and NLR were 97.56% [95%CI, 0.85 to 1.00], 69.23% [95%CI, 0.39 to 0.90], and 90.9% [95%CI, 0.77 to 0.97], 3.17 [95%CI, 1.40 to 7.18] and 0.03 [95%CI, 0.00 to 0.26], respectively. For the D920 respirator, pass rates were 92.59% [95%CI, 0.82 to 0.97] and 94.44% [95%CI, 0.85 to 0.99] for the 2.5-min and 6-min protocols, respectively, with sensitivity, specificity, PPV, PLR and NLR of 100% [95%CI, 0.91 to 1.00], 75% [95%CI, 0.22 to 0.99], 94.4% [95%CI, 0.84 to 0.99], 4.00 [95%CI, 0.73 to 21.84] and 0.02 [95%CI, 0 to not estimable]. During CC, baseline seal integrity was achieved in 98.15% of participants. The D918 showed clinically significant leakage during (44.44%) and post-compression (29.63%), contrasting with D920’s maintained performance (*p* > 0.05). Multivariate analysis identified nasal width (OR 8.71 [95%CI, 2.74 to 31.8]) and auricular arc length (OR 0.78 [95%CI, 0.64 to 0.95]) as independent predictors of fit quality, demonstrating inverse and positive correlations with seal integrity, respectively.

**Conclusion:**

In this exploratory paired simulation study, the OSHA 2.5-min protocol showed preliminary agreement with the 6-min Chinese reference protocol for quantitative N95 respirator fit testing among Chinese healthcare workers, particularly for the D920 respirator. However, the study does not establish formal equivalence or non-inferiority. Larger multicenter studies with prespecified non-inferiority margins are needed before the rapid protocol can be recommended as a replacement for the standard 6-min protocol.

## Highlights

- The preliminary agreement observed between the 2.5-minute and 6-minute testing methods suggests a potential opportunity to enhance the efficiency of fit testing for healthcare workers during public health emergencies.
- However, as this was an exploratory study without prespecified non-inferiority margins, the rapid protocol should be considered a promising screening approach rather than a validated replacement for the standard 6-minute method.
- These findings support the need for streamlined testing procedures, but larger multicenter studies are required to confirm their applicability.

## Introduction

During respiratory virus outbreaks, the use of N95 respirators is mandated for all personnel, particularly healthcare workers, to mitigate transmission risks (1, 2). The protective efficacy of N95 masks is largely dependent on their fit, which refers to the degree of alignment with the unique facial contours of the wearer. Fit testing is the standard method for determining this alignment (3–5). The internationally recognized protocol for fit testing is the 8-step method established by the U. S. Occupational Safety and Health Administration (OSHA), which requires a total duration of 7 min and 15 s. This protocol includes the following steps: normal breathing, deep breathing, turning the head side to side, moving the head up and down, speaking, making facial expressions, bending over, and concluding with normal breathing again. In 2010, China introduced the national standard GB19083-2010, which was adapted from the OSHA protocol (6). This standard simplifies the procedure to a 6-step method by omitting the facial expression and bending over steps, while retaining the remaining six steps, with a total duration of 6 min. This 6-step method has since been adopted as China’s substantial standard procedure, effectively replacing the OSHA 8-step method.

**Figure 1:**
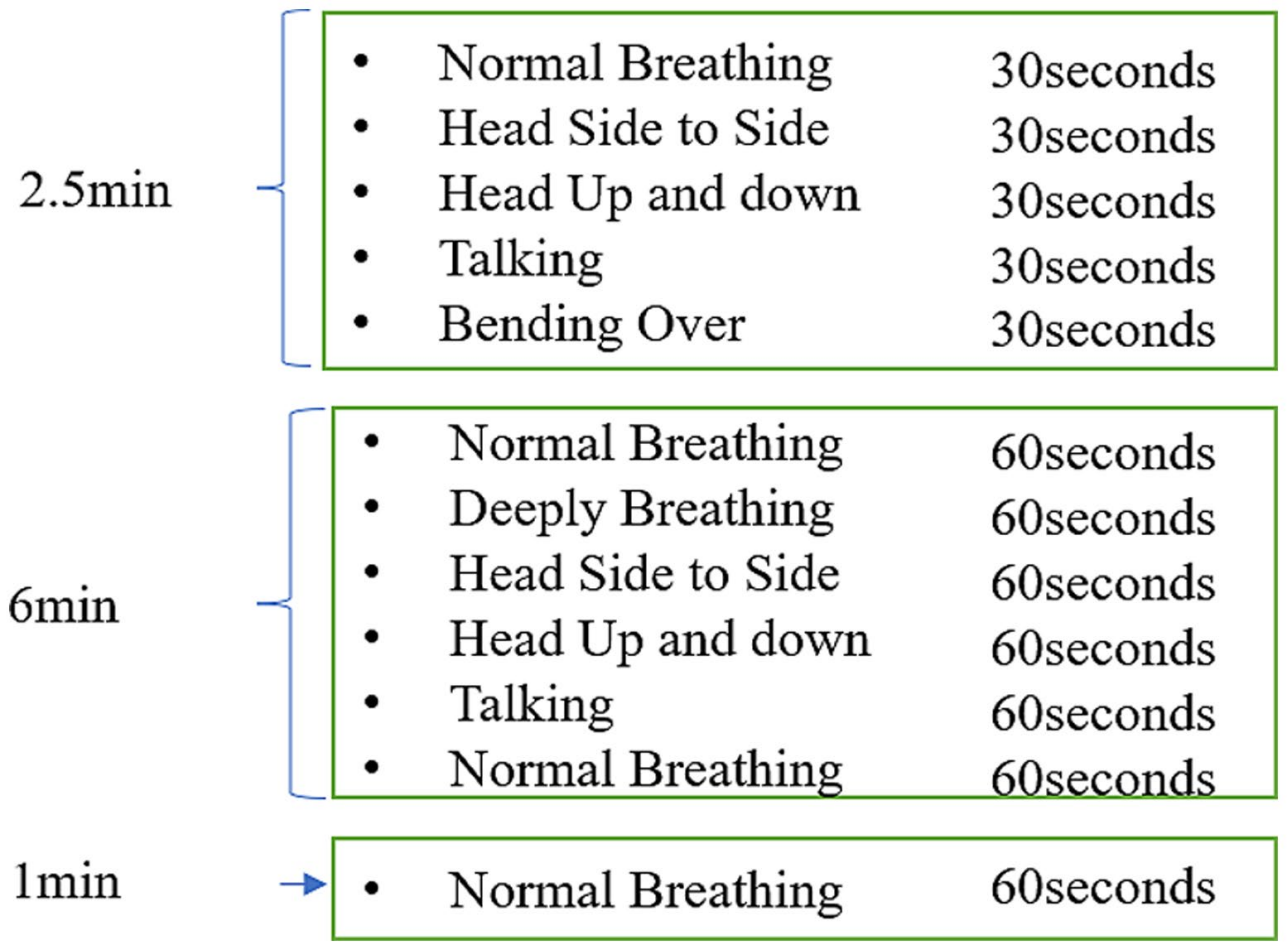
Mask fit test procedures.

In 2019, OSHA streamlined the 8-step fit-testing protocol through rigorous evaluation, introducing a rapid testing procedure substantial to four steps, with a total duration of 2.5 min. This simplified method includes bending over, speaking, turning the head side to side, and moving the head up and down. This revised protocol has been formally incorporated into the Code of Federal Regulations. Prior to the COVID-19 pandemic, OSHA estimated that this reduction in fit-test time by 5 min would save approximately 100,000 h of employee time annually, based on an assumption of 1.3 million respirator users (7). Currently, China lacks a corresponding rapid testing procedure. Therefore, the primary objective of this study is to evaluate whether the OSHA rapid testing protocol is substantial to China’s GB 19083–2010 standard (Figure 1).

Chest compressions during CPR can disrupt the respirator-face seal interface biomechanically, significantly reducing the protective efficacy of N95 respirators during resuscitation. Hyungoo Shin et al. (8) shows that different masks have different protective efficiencies during chest compressions (CC), with folded masks outperforming cup-shaped and valved respirators.

Proper mask usage is essential for respiratory medical staff to ensure effective protection. Implementing a rapid testing protocol work initiation can significantly enhance the efficiency of fit testing for medical personnel during public health emergencies, thereby reducing the response time of healthcare institutions. Currently, there is a lack of standardized rapid detection methods in China. In this study, we compared the 2.5-min testing method recommended by the U. S. Occupational Safety and Health Administration (OSHA) with the 6-min method outlined in the national standard (GB19083-2010). Additionally, we investigated mask leakage during chest compressions (CC) to assess the safety of N95 masks for bio-protection in clinical practice. This research aims to provide evidence-based recommendations for optimizing mask fit testing protocols in clinical settings.

## Method

### Study sites and institutional review boards

#### Design and oversight

**Figure 2:**
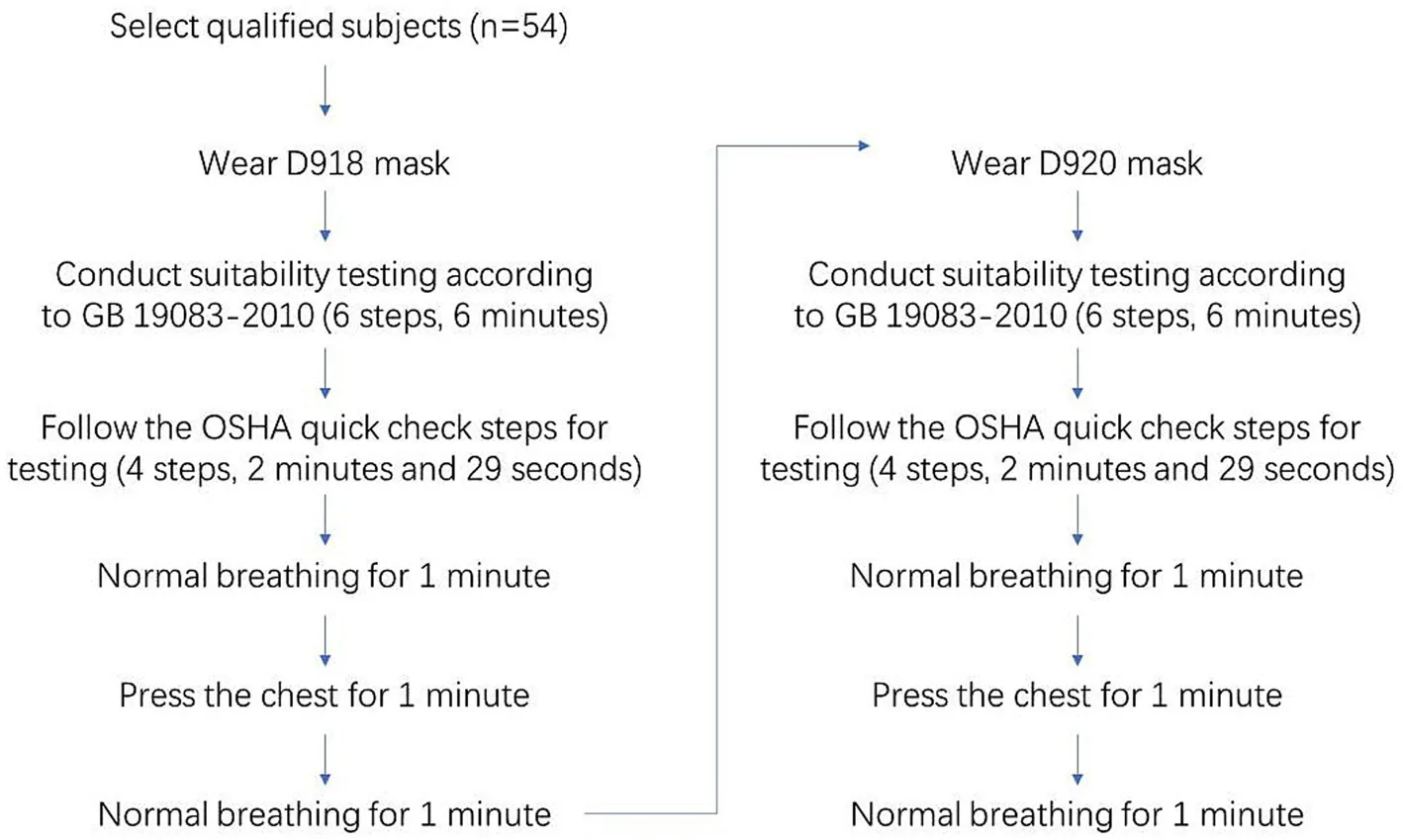
Operating procedures.

This study utilized a Paired Crossover Simulation and Diagnostic Agreement Study. A cohort of 54 medical staff members from our institution was recruited to wear two distinct types of medical masks, D918 and D920, undergoing a total of 540 mask fit tests. The study aimed to evaluate the practicality of different testing methods in routine clinical practice from two perspectives and to compare the effects of high-intensity clinical procedures, specifically cardiopulmonary resuscitation (CPR), on mask fit integrity. Using the six-step method (6 min) specified in the GB 19083–2010 standard as the reference standard, the study assessed the efficacy of the OSHA 2.5-min rapid test procedure within the Chinese healthcare worker population. The specific motion of chest compressions (CC) was selected as the primary focus of the investigation. The TSI PortaCount 8,048 was employed to monitor fit changes in the D918 and D920 masks before, during, and after CC. Both fit tests and anthropometric measurements of head and facial dimensions were conducted by the same examiner, ensuring a measurement error margin of no more than 5 mm. This approach ensured methodological consistency and minimized variability in data collection. The flowchart is shown in Figure 2. The order in which each participant wears the two types of masks is determined by a random number table generated by a computer.

#### Participants and setting

The tests were conducted in a controlled environment within a respiratory ward. The study participants consisted of healthcare workers. Exclusion criteria included: (1) known hypersensitivity or allergic reactions to medical masks, (2) pregnancy, (3) medical history of asthma, tuberculosis, bronchiectasis, lung cancer, or other relevant respiratory conditions. The study collected individual demographic and anthropometric factors that could potentially influence the fit of N95 masks, including facial measurements (e.g., nasal width, face length), age, height, weight, and gender. These variables were recorded to assess their potential impact on mask fit performance and to ensure a comprehensive analysis of influencing factors.

#### Procedures, interventions, and group allocation

**Figure 3:**
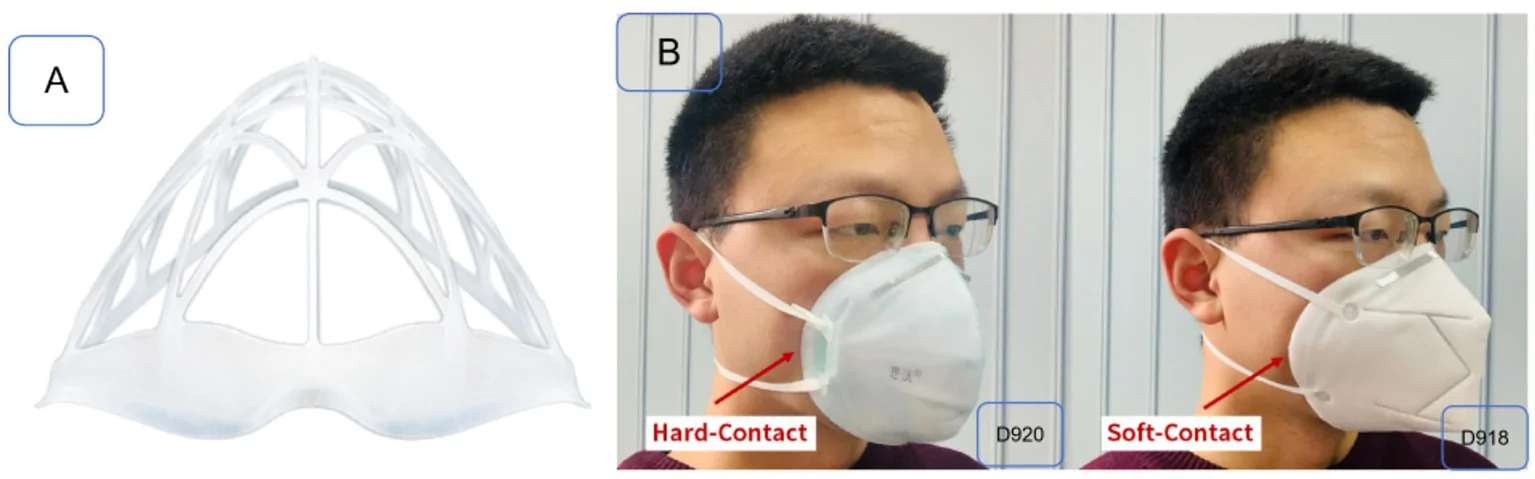
Two types of foldable face masks. **(A)** The frame structure of the D920 mask; **(B)** D920 mask-cup-shaped type with support frame and D918 mask-foldable flat-fold type.

**Figure 4:**
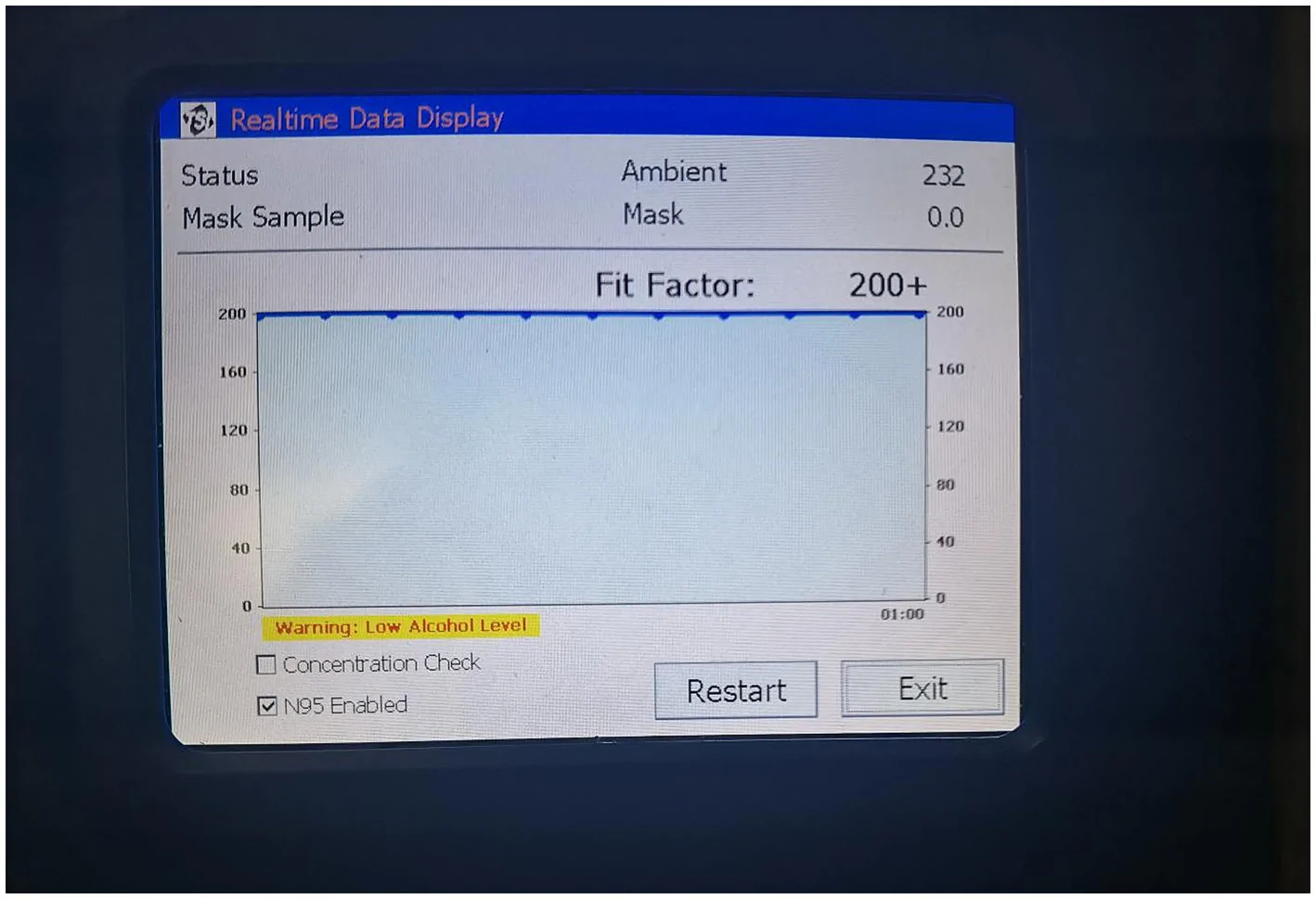
Real-time data display.

The CPR Anne mannequin (Laerdal Medical, Stavanger, Norway) served as the simulation tool, Frequency: A metronome is used for guidance to maintain a rate of 100–120 compressions per minute; Depth: 5–6 centimeters, with real-time visual feedback provided by the built-in pressure sensor of the manikin; Posture: Kneel on the right side of the manikin, keep both elbows fully extended, and align the shoulders vertically above the sternum; Personnel qualifications: All participants are medical staff certified in Basic Life Support (BLS). In addition, regarding the traction interference that PortaCount sampling tubes may exert on mask seal integrity, we have highlighted this as the primary technical limitation in the discussion and recommended that future studies adopt wireless or lightweight sampling equipment. The TSI PortaCount 8,048 was utilized as the testing device. The N95 masks evaluated in this study included the D920 (cup-type) and D918 (folded-type), both manufactured by SINOVO (Figure 3). After completing the initial fit test, participants were instructed to perform continuous chest compressions (CC) on the CPR Anne mannequin for 1 min while proceeding to the subsequent test phase, without touching or adjusting the mask. If the mask straps were observed to be loose, participants were required to readjust them and restart the test. Healthcare workers (HCWs) participated in a crossover fit-testing protocol. All participants were required to wear the masks in real-time before advancing to the next test, as depicted in Figure 4. All mask test reports confirmed a filtration efficiency exceeding 98%, ensuring compliance with regulatory standards for respiratory protection.

#### Fit testing

N95 testing mode, Fit Factor (FF) values range from 1 to 200. According to OSHA 29 CFR 1910.134 and GB19083-2010, an FF value of ≥100 is acceptable, while an FF value of <100 is unacceptable (6).

### Statistical analyses

This was designed as a pilot simulation study, and the sample size (*n* = 54) was determined based on practical feasibility and recruitment capacity within the study period, rather than through formal power calculation. Sample size calculations were not performed. The Shapiro–Wilk test was used to assess the normality of continuous variables (including log-transformed fit factor values). As the data were not normally distributed, non-parametric tests were employed for paired comparisons. For paired binary outcomes (pass/fail): McNemar’s test. For paired continuous outcomes (fit factor values): Wilcoxon signed-rank test. For agreement between the two protocols: Cohen’s Kappa coefficient with 95% confidence intervals. For diagnostic performance: Sensitivity, specificity, predictive values, and likelihood ratios with 95% CIs, based on 2 × 2 contingency tables. Multivariable analysis: Dependent variable: Pass/fail outcome of the 2.5-min test. Independent variables: Age, sex, BMI, nasal width, ear arc length, mask type (D918 vs. D920), and baseline fit factor (6-min test result). Variable selection: We used a backward stepwise elimination approach, with a retention criterion of *p* < 0.10. Results are reported as adjusted odds ratios (aOR) with 95% confidence intervals. *Post-hoc* analyses utilized the Wilcoxon rank-sum test for non-parametric data and a Bonferroni-corrected paired test for parametric data. A *p*-value < 0.05 was deemed statistically significant. Statistical analyses were conducted using SPSS 28.0.

## Result

All 54 participants completed the study. 10 (18.5%) were male, with a median age of 27 years (IQR, 25–30). The majority of participants were nurses (81.5%, *n* = 44) with a normal body mass index. The 6-min test results were used as the reference standard (Table 1).

**Table 1:** Demographics.

Variables	*N* = 54
Sex, male (%)	10(18.5%)
Age, yr. (median, IQRs)	27 (25–30)
Occupation, (%)	
Nurse	44 (81.5%)
Physicians	10 (18.5%)
Body mass index, BMI (%)	
Underweight	10 (18.5%)
Normal	33 (61.1%)
Overweight	11 (20.4%)
Subjective fit score	
D918 mask	10 [8, 13]
D920 mask	9 [8, 10]

### Differences in mask fit test results across different durations

#### D918 mask

During the 2.5-min test, the Fit Factor (FF) values ranged from 66 to 200, with a pass rate of 75.93% [95%CI, 0.63 to 0.85]. In the 6-min test, FF values ranged from 81 to over 200, yielding a pass rate of 81.48% ([95%CI, 0.69 to 0.90], *p* = 0.371). The results demonstrated good consistency, with a kappa (K) value of 0.725 (Table 2).

**Table 2:** Fast test (1 min and 2.5 min) vs. Chinese standard testing procedures (6 min).

Mask type/Parameter	1 min fast test	2.5 min test	6 min test	*p* value (2.5 vs 6)	*p* value (1 vs 6)	*p* value (1 vs 2.5)
D918 mask (*n* = 54)						
Total FF	200[66, 200]	154[65, 200]	163[81, 200]			
Passing Rate, n (%)	53(98.15%)	44(81.48% [95%CI, 0.69 to 0.90])	41(75.93% [95%CI, 0.63 to 0.85])	0.371	0.003	0.016
Failed FF	66[66, 66]	76[65, 94]	93[81, 98]			
D920 mask (*n* = 54)						
Total FF	200[17, 200]	200[58, 200]	200[116, 200]			
Passing Rate, n (%)	52(96.30%)	51(94.44% [95%CI, 0.85 to 0.99])	50(92.59% [95%CI, 0.82 to 0.97])	1.0	0.480	1.0
Failed FF	48[17, 79]	58[58, 58]	–^†^			

FF = Median [min, max], each participant was tested twice, resulting in a total of 108 observations across the two mask types.

^†^Four participants failed the 6‑min test with the D920 mask; their specific Fit Factor values were not recorded by the device.

#### D920 mask

For the 2.5-min test, FF values ranged from 58 to 200, while for the 6-min test, FF values ranged from 116 to 200. The pass rates were 92.59% [95%CI, 0.82 to 0.97] and 94.44% [95%CI, 0.85 to 0.99], respectively (*p* = 1.000). The consistency was excellent, with a kappa (K) value of 0.847.

The sensitivity and specificity of the user seal check for identifying actual gross leakage were approximately 97.56% [95%CI, 0.85 to 1.00] and 69.23% [95%CI, 0.39 to 0.90] for the 2.5-min test with the D918 mask, and 100% [95%CI, 0.91 to 1.00] and 75% [95%CI, 0.22 to 0.99] for the D920 mask, respectively. The Positive Predictive Value (PPV) was 90.9% [95%CI, 0.77 to 0.97] for the D918 mask and 94.4% [95%CI, 0.84 to 0.99] for the D920 mask. The positive likelihood ratio (PLR) of the D918 mask is 3.17 [95%CI, 1.40 to 7.18], and the negative likelihood ratio (NLR) is 0.03 [95%CI, 0.00 to 0.26]; the D920 mask exhibits a positive likelihood ratio (PLR) of 4.00 [95%CI, 0.73 to 21.84] and a negative likelihood ratio (NLR) of 0.02 [95%CI, 0 to not estimable].

### Protective performance in CC

#### D918 mask

Before CC, the leakage rate was 1.85%. During CC, the leakage occurred in 24 individuals, dropping the pass rate to 55.56%. After CC, the pass rate increased to 70.37% (*p* < 0.05; Table 3).

**Table 3:** Effect of chest compressions on mask fit.

Mask type	Time point	Passed (n)	Not Passed (n)	Passing rate (%)	*p* value
D918	Before CC	53	1	98.15%	< 0.001^#^ 0.043^※^ 0.001^*^
	During CC	30	24	55.56%	
	After CC	38	16	70.37%	
	Total	130	32		
D920	Before CC	52	2	96.30%	0.480^#^
	CC	50	4	92.59%	0.617^※^
	After CC	52	2	96.30%	0.480^*^
	Total	154	8		

# Comparison between Before CC and During CC. ※Comparison During CC and After CC. *Comparison between Before CC and After CC.

#### D920 mask

The pass rates were 96.30% before CC, 92.59% during CC, and 96.30% after CC, with no statistically significant differences among the rates, *p* > 0.05.

### Facial factors

The width of the nasal bridge was identified as a significant risk factor for mask leakage, with broader nasal dimensions associated with a poorer mask seal. The adjusted odds ratio (OR) for this association was 8.71 [95% confidence interval (CI), 2.74 to 31.80], indicating a strong positive correlation. In contrast, the curvature length of the ear was found to be a protective factor, as longer ear curvatures were linked to improved mask fit. The adjusted OR for this protective effect was 0.77 [95% CI, 0.64 to 0.95], suggesting a moderate inverse relationship. These findings highlight the importance of facial anatomical variations in determining mask fit efficacy.

### Adverse events

**Figure 5:**
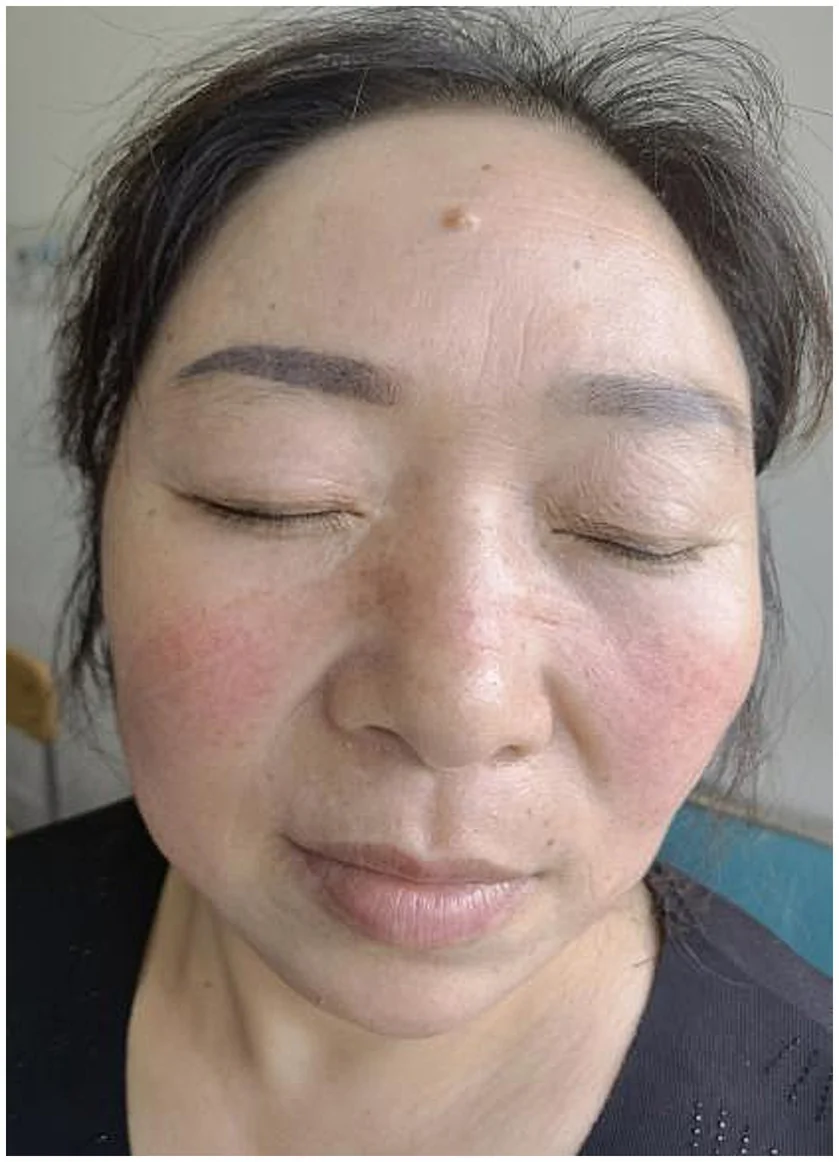
Facial depression.

All subjects had mild facial depression (Figure 5).

## Discussion

We performed paired tests to assess the robustness and consistency of our findings. The use of identical standardized testing equipment across all sessions ensured the reliability and reproducibility of the results. All participants were equipped with N95 masks and underwent a real-time fit assessment before each test session to verify proper mask placement and seal integrity. This protocol was implemented to minimize variability and ensure the accuracy of the data collected.

The 2.5-min rapid test showed a high sensitivity of 97.56% for the folded mask (D918) and 100% for the cup-shaped mask (D920). However, the specificities were modest, at 69.23 and 75%, respectively, indicating that the rapid test is more reliable for ruling out poor fit than for confirming good fit. Our findings diverge from those reported by Regli et al. (9), which may be attributed to the superior fit and high pass rates of the masks utilized in our study. According to the test report provided by SINOVO, the filtration efficiency of the masks exceeded or approached 99%. However, further validation of our results is warranted through larger-scale studies involving masks with varying degrees of fit to ensure broader applicability and generalizability. The rapid 2.5-min protocol demonstrated excellent negative likelihood ratios (LR- < 0.1) for both respirator types, suggesting it is highly reliable for excluding poorly fitting respirators. However, the moderate positive likelihood ratios (LR + = 3–4) indicate that passing the rapid test provides only modest confirmation of true compliance with the standard 6-min reference, particularly for the foldable D920 model (LR + = 4).

We further investigated the efficacy of rapid testing methods in specialized environments and operational scenarios for healthcare workers (HCWs) by simulating chest compressions (CC) maneuvers on a mannequin. Current international CPR guidelines do not specify the level of airway protection required for HCWs when performing CPR on patients suspected or confirmed to have airborne infectious diseases (10, 11). In our study, significant differences were observed in the pass rates of participants wearing the D918 and D920 masks before, during, and after CC. The D918 mask exhibited a pattern of initial decline followed by recovery, suggesting that dynamic clinical activities such as CC significantly influence mask leakage (10). The study by Hwang et al. (10) demonstrated that N95 respirators fail to provide adequate respiratory protection during CPR-related chest compressions, particularly among participants who failed the initial fit testing. During CC, the operator’s head undergoes substantial and frequent movements, altering the relative position of the mask on the face and leading to compromised seal integrity. As a result, the pass rate for the D918 mask decreased from 98.15 to 55.56%, aligning with findings reported by Sung Yeon Hwang (10). Given the pronounced pulling effect of the sampling tube on the mask during CC, the actual pass rate is likely higher than 55.56%. These findings indicate that CC exerts a measurable impact on the seal integrity of N95 masks. It is important to note that a respirator can only function effectively when a secure facial seal is maintained (2). Following CC, with the cessation of risk factors and ongoing mask deformation, the pass rate rebounded to 70.37%. To mitigate infection risks, it is critical for wearers to receive proper training on mask fitting before entering environments with potential infectious exposure (2, 10) and to make timely adjustments to their masks as needed.

Hyungoo Shin (8) demonstrated that the physical properties of respirators can differentially influence their protective performance during chest compressions (CC). The D920 mask, a specially engineered support-frame respirator, maintained a consistently high pass rate (exceeding 92.5%) across all three phases of CC. Designed to accommodate the facial anatomy of East Asians, the mask incorporates a ribbed support structure on its cover, ensuring firm contact between the mask’s edge and the face. This design minimizes the relative displacement of the mask during movement, thereby preserving its protective efficacy throughout the CC process. Suen et al. (12) employed a portable aerosol spectrometer to evaluate the performance of N95 respirators during various nursing procedures, such as nebulizer inhalation and nasogastric tube insertion. Their findings (12) revealed a significant decline in the average Fit Factor (FF) from 184.85 to 134.71 post-procedure, with 33% of participants exhibiting an FF below 100. In our study, the pass rate for healthcare workers wearing the D918 mask during a standard 6-min test was 75.93%, whereas only 55.56% achieved protection during CC, highlighting a statistically significant discrepancy. This observation underscores that passing the conventional 6-min test does not necessarily ensure protection against infection risks during other clinical interventions.

Healthcare workers may necessitate the use of specific types of N95 respirators or more rigorous mask-fit testing protocols, such as incorporating dynamic actions like CC as supplementary measures to standard testing procedures (8, 13–15). However, further large-scale studies are required to validate whether the 2.5-min testing method can be universally applied as an effective mask-fit assessment tool for healthcare workers in specialized operational environments. Particularly for high-risk clinical procedures, it may be essential to simulate these actions to accurately evaluate the associated infection control risks (12).

To mitigate mask leakage, healthcare workers often tighten the straps, which can result in facial pressure injuries (3, 14). Additionally, our study identified that a wider nasal bridge is a risk factor for compromised mask fit, while the length of the lower curvature of both ears serves as a protective factor (16). These findings highlight the importance of considering individual anatomical variations when optimizing mask fit and minimizing leakage risks. There is a significant difference in the pass rate of N95 respirators between different brands/models, which may be related to the design of facial fit (17). The wider nasal breadth may create gaps at the nasolabial fold region, while longer ear arc length alters the positioning and tension of the head straps, affecting the vector of compressive force around the cheek-seal interface—particularly during dynamic jaw movements associated with chest compressions. The current international respirator fit-test panel, established by Los Alamos National Laboratory (LANL) in 1978 (18), demonstrates limited applicability to Chinese populations due to inherent craniofacial morphological disparities. Comparative anthropometric analyses of 3,000 Chinese civilian respirator users revealed statistically significant ethnic variations, including shorter face length, smaller nose protrusion, larger face width, and longer lip length compared to Western cohorts (*p* < 0.001) (19). Prior studies have shown that Chinese and other Asian populations differ from U. S./Western respirator fit-test panels in multiple craniofacial dimensions, including facial width, nasal dimensions, nose protrusion, and bitragion-related arc measurements (20–23). These population-specific differences may influence respirator fit performance and limit the direct generalizability of fit data derived from Western populations, supporting the need for region-specific respirator design and fit-test standards (24, 25). 86% fit-test failure rate among South African subjects using LANL-derived medium-sized P2 respirators (26). Han et al.’s call for ethnically tailored filtering facepiece respirator (FFR) designs in Korea further corroborate the necessity of population-specific respiratory protection solutions (27). Systematic evaluation of FFR facial interface biomechanics across diverse demographic groups is therefore imperative to optimize personal protective equipment efficacy. We now explicitly suggest that respirator manufacturers should consider producing multiple size variations with adjustable strap configurations to accommodate the variability in nasal width and ear arc length among Asian end-users. We also recommend that occupational health practitioners consider these simple anthropometric measurements as low-cost screening tools during respirator selection.

As a standard component of their pre-shift preparations, these workers perform mask fit tests to ensure optimal protection and compliance with safety protocols (28). Public health authorities and policymakers should carefully consider our findings when selecting appropriate testing methodologies for hospital respiratory protection programs, particularly for healthcare workers who face elevated risks of exposure to airborne pathogens (29–31).

## Conclusion

This exploratory paired crossover simulation study suggests that the OSHA 2.5-min quantitative fit-testing protocol may provide preliminary agreement with the 6-min Chinese reference protocol among Chinese healthcare workers, especially for the D920 respirator. However, the study was limited by its small sample size, lack of prespecified non-inferiority margin, and single-center simulation design. Therefore, the 2.5-min protocol should be considered a promising rapid screening approach rather than a validated replacement for the standard 6-min protocol. Larger multicenter studies are needed to determine whether the rapid protocol can be adopted in routine occupational-health practice.

Limitation: In this study, we state that only two commercially available N95 models (one foldable, one cup-shaped) were tested, and the findings may not be generalizable to other respirator designs or brands. The modest sample size may affect statistical power, particularly for subgroup analyses and multivariable logistic regression, and we note that the findings should be interpreted as preliminary evidence requiring validation in larger cohorts. Although we identified nasal width and ear arc length as predictors of fit quality in this exploratory analysis, our assessment of facial anatomical variations was not exhaustive. Other potential craniofacial dimensions—such as face length, nose protrusion, bigonial breadth, and bitragion submandibular arc—were not systematically evaluated. Future studies with a broader anthropometric panel and larger sample sizes are needed to comprehensively delineate the relationship between facial morphology and respirator seal integrity. We emphasize that chest compressions performed on a manikin in a controlled laboratory setting may not fully replicate the physical exertion, sweat accumulation, body movements, and prolonged wear time encountered in actual clinical resuscitation scenarios. We also note that simulated CPR may differ from real-world CPR in terms of compression depth consistency, feedback device usage, and the psychological stress component, all of which could affect mask seal integrity.

## Data Availability

The raw data supporting the conclusions of this article will be made available by the authors, without undue reservation.
