# Correction: Evaluating a rapid fit-testing method for N95 respirators in Chinese healthcare workers: a paired crossover simulation and diagnostic agreement study

**DOI:** 10.3389/fpubh.2026.1957646

**Published:** 2026-09-09

**Authors:** Guo Jing Han, Lina Li, Ru Fan, Wei Li, Zhao Jun Sheng, Wei Hua Zhang

**Affiliations:** 1 Department of Respiratory and Critical Care Medicine, the First Medical Centre of Chinese PLA General Hospital, Beijing, China; 2 Beijing SINOVO Times Technology Co. Ltd., Beijing, China; 3 Department of Respiratory and Critical Care Medicine, the Eighth Medical Center of Chinese PLA General Hospital, Beijing, China

**Keywords:** chest compressions, fit testing, healthcare workers, N95 respirators, rapid-test protocol

An incorrect **Funding** statement was provided.

The funder Randomized Controlled Trial of Phage Cocktail Therapy for Ventilator-Associated Pneumonia Caused by Drug-Resistant Gram-Negative Bacteria 2025YFC3408504 to Weihua Zhang was erroneously omitted.

The funder Research and Development of Safe and Efficient Phage Preparations and Key Technologies for Clinical Treatment of Drug-Resistant Bacterial Infections ID: 2025YFC3408504 was erroneously included.

The correct **Funding** statement reads:

“The author(s) declared that financial support was received for this work and/or its publication. This work was supported by the subproject Randomized Controlled Trial of Phage Cocktail Therapy for Ventilator-Associated Pneumonia Caused by Drug-Resistant Gram-Negative Bacteria 2025YFC3408504, affiliated to the parent project Research and Development of Safe and Efficient Phage Preparations and Key Technologies for Clinical Treatment of Drug-Resistant Bacterial Infections to WZ.”

The original version of this article has been updated.

